# Pulsed Electrical Stimulation Enhances Consistency of Directional Migration of Adipose-Derived Stem Cells

**DOI:** 10.3390/cells10112846

**Published:** 2021-10-22

**Authors:** Mi Hee Lee, Ye Jin Park, Seung Hee Hong, Min-Ah Koo, Minyoung Cho, Jong-Chul Park

**Affiliations:** 1 Cellbiocontrol Laboratory, Department of Medical Engineering, Yonsei University College of Medicine, Seoul 03722, Korea; leemh1541@yuhs.ac (M.H.L.); yejin3147@gmail.com (Y.J.P.); seungheestar@yuhs.ac (S.H.H.); drmin9@yuhs.ac (M.-A.K.); bancrose@naver.com (M.C.); 2 Department of Medical Device Engineering and Management, Yonsei University College of Medicine, Seoul 03722, Korea; 3 Department of Medical Engineering, Graduate School of Medical Science, Brain Korea 21 Project, Yonsei University College of Medicine, Seoul 03722, Korea

**Keywords:** electrotaxis, directional migration, adipose-derived stem cells, Golgi polarization

## Abstract

Electrical stimulation is a well-known strategy for regulating cell behavior, both in pathological and physiological processes such as wound healing, tissue regeneration, and embryonic development. Electrotaxis is the directional migration of cells toward the cathode or anode when subjected to electrical stimulation. In this study, we investigated the conditions for enhanced directional migration of electrically stimulated adipose-derived stem cells (ADSCs) during prolonged culture, using a customized agar-salt electrotaxis chamber. Exposure of ADSCs to a 1200 μA electric current for 3 h, followed by cessation of stimulation for 6 h and resumed stimulation for a further 3 h, increased directional cell migration toward the anode without inducing cell death. Moreover, Golgi polarization maintained the direction of polarity parallel to the direction of cell movement. Herein, we demonstrated that a pulsed electric current is sufficient to trigger directional migration of ADSCs in long-term culture while maintaining cell viability.

## 1. Introduction

Cell migration is a fundamental process whereby cells are able to alter their position and move to the appropriate locations in order to execute their biological functions. This process is essential for proper development and maintenance of bodily tissues, as well as for the success of cell therapy [1,2,3]. In an adult organism, cell migration plays a central role in many physiological and pathological phenomena, including immune response, wound repair, vascular diseases, and cancer metastasis [4,5].

In recent decades, many studies have demonstrated that stem cell therapy could be used as an attractive therapeutic approach to prevent and treat numerous diseases. Multiple cell types can be utilized in such therapies, including primary, progenitor and stem cells [6,7]. Adipose-derived stem cells (ADSCs) can be obtained by subcutaneous liposuction, which requires a less invasive procedure and minimizes donor site morbidity and patient discomfort. In addition, as ADSCs are located in adipose tissue and present no ethical and political issues compared to embryonic stem cells. Moreover, ADSCs, compared to MSCs from other sources, have the highest proliferation ability, and retain their pluripotency after a long culture period. These characteristics make ADSCs a more acceptable solution for tissue engineering applications in the field of regenerative medicine and clinical studies [8,9,10].

Endogenous electrical signals are known to induce cellular events such as proliferation, tissue regeneration, embryonic development, and wound healing, in vivo. Several studies have reported that electrical stimulation (ES) is an important cue that guides cell behavior and regulates directional cell movement, both in vivo and in vitro, in response to physical stimulation [11,12,13,14]. The use of bioelectricity plays an important role in human regeneration and there are several reports of continuous ES inducing wound healing [15], bone regeneration [16,17,18], chondrogenesis [19], and the activation of rapid action potential in nerve and muscle cells [20]. Applied direct electric current can induce the directional migration of many cell types, including endothelial cells, bone marrow mesenchymal stem cells, and human dermal fibroblasts; this phenomenon is known as electrotaxis. During electrotaxis, cells subjected to direct electric current will move preferentially toward either the anode or cathode, indicating that the effects of electric signals on cells are specific to different cell types and species [21,22,23]. Researchers have previously demonstrated that exogenous direct ES can effectively guide directional migration toward the anode of ADSCs, human mesenchymal stem cells (hMSCs), and tonsil derived MSCs [24,25,26]. In our previous studies, we showed that cells migrate toward the cathode or anode following Golgi apparatus (GA) polarization, in the same direction as the ES. Therefore, GA polarization is considered a predominant stimulus in directed cell migration induced by electric treatment [27,28].

Although ES induces a directional cell-response, stronger currents and longer exposure times can result in decreased cell viability due to an electrochemical reaction in the medium [29,30]. The aim of this study was to determine the ideal ES conditions to guide and maintain directional migration in adipose-derived stem cells without inducing cell death during prolonged culture.

## 2. Materials and Methods

### 2.1. Electrical Stimulation of ADSCs to Induce Electrotaxis

**Figure 1:**
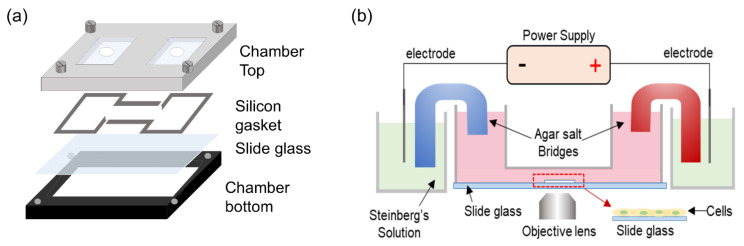
Schematic illustration of the customized agar-salt electrotaxis chamber and system. (**a**) Deconstructed view of electrotaxis chamber used for real-time observation of migration of cells cultured on a glass slide. (**b**) The chamber was installed on the incubator system, which consisted of a CO_2_ and temperature-controlled chamber with reservoirs connected to the wells at each end of the chamber via 2% agar-salt bridges. Electrical stimulation was applied through platinum electrodes immersed in Steinberg’s solution contained in the reservoirs. Cell behavior was observed using an inverted microscope and recorded using a CCD camera connected to a personal computer.

A customized agar-salt electrotaxis chamber with an incubator system was used to generate ES. Briefly, electrotaxis analysis was performed on a stage top incubator system using CCP ver. 3.8 software (Live Cell Instrument, Inc., Seoul, Korea), and equipped with an inverted microscope (Olympus Corporation, Tokyo, Japan). The electrotaxis chamber consisted of a chamber base on which a cell-seeded glass slide was placed, followed by a silicon gasket and finally the chamber top, connected by two 2% agar-salt bridges in Steinberg’s solution (Figure 1) [24]. Human ADSCs (Lonza, Basel, Switzerland) were cultured in ADSC growth medium (ADSC-GM, Lonza) at 37 °C in a 5% CO_2_ environment. Cells from passages 4–7 were used in all experiments. The ADSCs were seeded at a density of 1 × 10^4^ cells/mL on a glass slide with a silicon O-ring of 6 mm inner diameter and incubated for 16–24 h in the CO_2_ incubator. The cell-seeded glass slide was placed on the chamber base, followed by the silicon gasket and chamber top placed over the slide. Thereafter, an electric current was applied through platinum electrodes immersed in reservoirs containing Steinberg’s solution, which were connected to the wells at each end of the chamber via 2% agar-salt bridges. The electrotaxis chambers containing the culture medium were exposed to constant ES at 1200 μA for 3 h or pulsed ES. During the experiment, electricity flow was measured using a digital power meter to ensure consistency of the electric current.

### 2.2. Measurement of ADSC Migration

Following ES, cell migration speed and X-directedness were calculated using the Manual Tracking, and Chemotaxis and Migration Tool plug-ins of ImageJ 1.37v software (ImageJ, National Institutes of Health, Bethesda, MD, USA) from cell images captured every 5 min using a CCD camera connected to a personal computer. The center of each nucleus was manually selected at each time point. The migration speed was calculated as the displacement distance of a cell along the X-axis, divided by the time required for the displacement to occur. X-directedness was evaluated toward either the cathode or anode; a cell moving parallel to the current toward the anode would have a directedness of 1, whereas a cell moving directly toward the cathode would have a directedness of −1. A directedness value close to 0 represents random movement of cells. For migration analysis, more than 50 cells were randomly selected from at least 3 independent experiments. Mitotic and dying cells, as well as those migrating outside of the field of view, were excluded from the analysis.

### 2.3. Cell Viability Assay

Cells were seeded in the center of a 2-well glass slide at a density of 1 × 10^5^ cells/mL and allowed to adhere overnight. The prepared cells were then subjected to ES for 3, 6, and 9 h or pulsed conditioning, respectively. Cell viability after to ES was assessed using the 3-(4,5-dimethylthiazol-2-yl)-2,5-diphenyltetrazolium bromide (MTT; Amresco, Solon, OH, USA) and Live/Dead (Molecular Probes, Eugene, OR, USA) assays. All experiments were repeated in triplicate and results presented as mean ± standard deviation.

### 2.4. Immunofluorescence Staining and Golgi Polarization Analysis

Actin cytoskeletons, GA, and nuclei were visualized using immunofluorescence staining subsequent to 3 h or pulsed ES. The electrically treated cells were fixed with 4% paraformaldehyde for 15 min at room temperature and then washed twice with Dulbecco’s phosphate-buffered saline (DPBS). Cells were permeabilized with 0.1% Triton X-100 in DPBS for 5 min at RT and then washed three times with DPBS for 5 min. Bovine serum albumin (1%; BSA) was added and left to incubate for 30 min at RT to block nonspecific binding. The cells were then incubated with a GM130 primary antibody (dilution 1:250; Abcam, Cambridge, UK) and treated with Alexa Fluor^®^ 488 phalloidin (5U/mL; A12379, Invitrogen, Carlsbad, CA, USA), at 4 °C overnight to label the actin cytoskeleton. Cells were then rinsed at least three times with DPBS and treated with goat anti-Rabbit IgG conjugated with Texas Red (dilution 1:1000; T-2767, Invitrogen) for 1 h at RT in the dark to label GM130. After DPBS washing, cells were treated with Hoechst #33258 (B2883, Sigma-Aldrich, St. Louis, MO, USA) for 10 min at RT in the dark before being rinsed twice with DPBS. Fluorescence images were acquired using an LSM700 confocal fluorescence microscope (Carl Zeiss, White Plains, NY, USA).

### 2.5. Statistical Analysis

Data were expressed as means ± standard error of the mean (SEM). All statistical analyses were performed using SPSS Version 23.0 software (IBM Corp., Armonk, NY, USA) and means were compared using one-way analyses of variance (ANOVA). A value of *p* < 0.05 was considered statistically significant.

## 3. Results and Discussion

**Figure 2:**
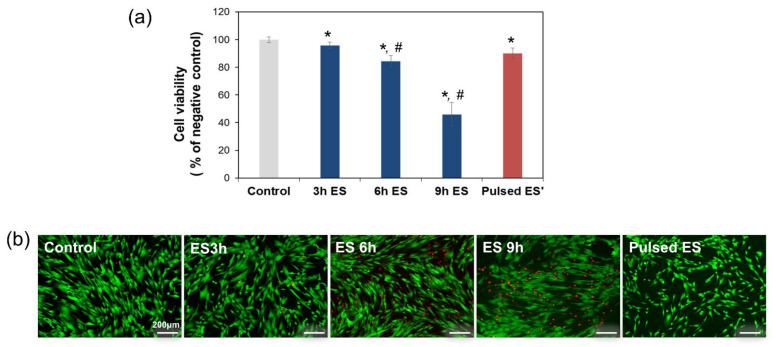
Effect of electrical stimulation (ES) on cell viability, as measured by the MTT assay (**a**) and Live/Dead assay (**b**) after 1200 μA ES was applied for different prolonged exposure times. * represents *p* < 0.05 compared to controls not exposed to electric current. # represents *p* < 0.05 compared to cells subjected to 3 h ES.

We investigated cell damage caused by ES of ADSCs in accordance with electrical exposure time. At 18 h after cell seeding, ADSCs were subjected to 1200 μA ES as follows: continuous stimulation for periods of 3, 6, and 9 h, or pulsed stimulation respectively; controls were not exposed to any electrical signal. Figure 2a shows the percentage of surviving cells determined by MTT assay, following the different ES exposure modes. Compared with 100% in the control, cell viability decreased slightly to 95% after exposure to ES for 3 h. At an ES exposure time of 6 h, cell viability decreased below 80%, whereas the maximum decrease was observed at 9 h of ES. To evaluate whether pulsed ES would reduce cell damage, ADSCs were exposed to pulsed ES for 3 h, followed by 6 h resting time, and 3 h re-exposure. Less reduction in cell viability was observed in cells exposed to pulsed ES than in cells stimulated continuously for 6 h. Similar results were obtained from the outcomes of Live/Dead assay (Figure 2b). Similar to the control, no notable cell death was observed with pulsed ES, whereas prolonged exposure to ES increased the population of dead cells.

Previously reported studies confirmed that long-term ES can result in nutrient depletion and loss of ions from culture media, alongside electrochemical reaction in the medium and increased cell death [29,30]. Cells are known to intrinsically possess the mechanisms to accumulate the incremental effects of ES. Cell membrane permeability is altered by ES, resulting in apoptosis through several different intracellular molecular mechanisms, including the induction of reactive oxygen species by Ca^2+^ influx. Therefore, we predicted that ES caused membrane breakdown by exceeding the relevant transmembrane voltage threshold, although cell membranes did not rupture as seen in electroporation, where high voltage pulsed electric fields are applied for extremely short durations, such as nano- or picoseconds [31,32,33,34]. Furthermore, it has been reported that ES of excessive duration results in decreased expression of SIRT3 a member of the mammalian sirtuin protein family, which is located in the mitochondria and regulates energy balance, oxidative metabolism, oxidative stress, and cell damage and leads to loss of mitochondrial function [35]. However, the mechanisms by which cells are affected by prolonged ES remain fairly unclear.

The results of the cell viability assay indicated that ES induced cell damage in a time-dependent manner. However, we observed that the same amount of electric current applied in a pulsed manner revealed different results compared with continuous electrical exposure. When cells were exposed to repeated bursts of ES, the cell survival rate was higher than on exposure to uninterrupted ES. These results showed that the application of a sustained electric current had the potential disadvantage of cytotoxicity and that cell metabolic activity decreased considerably with an increase in ES exposure time.

**Figure 3:**
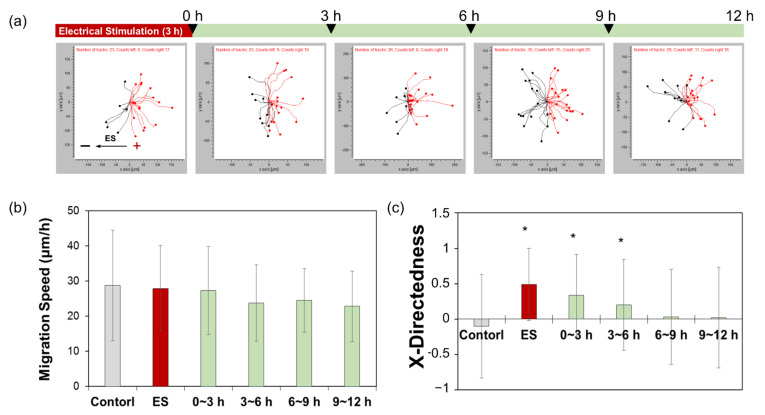
Migration assay of ADSCs subjected to single-round electrical stimulation (ES) for 3 h, followed by no stimulation. (**a**) Cell tracking data, (**b**) migration speed, and (**c**) X-directedness were determined for ADSCs subjected to 1200 μA ES for 3 h, followed by no ES for 12 h. * represents *p* < 0.05 compared to controls not exposed to electric current.

To determine the threshold values for inducing electrotaxis, ADSCs were exposed to 1200 μA ES for 3 h, after which the current was turned off. Cell migration behaviors were analyzed after 3 h ES, and at certain intervals for 12 h after the ES had ceased. In the absence of an electric current, no distinct directional migration by the ADSCs was observed at all-time points; the ADSCs merely migrated in random directions, as indicated by the migration trajectories (data not shown). However, in the presence of an electric current applied for 3 h, ADSCs showed significant directional migration toward the anode (Figure 3a). The speed of migration was not affected by the presence or absence of an electric current (Figure 3b). After the electric current had been switched off, the X-directedness gradually decreased. Nevertheless, the cells continued to move toward the anode for 6 h (Figure 3c). These results suggested that the directional migration induced by a single exposure to electric current could be maintained for 6 h. Therefore, we evaluated the potential to control cell migration through exposure to electric current by regulating the frequency of exposure.

**Figure 4:**
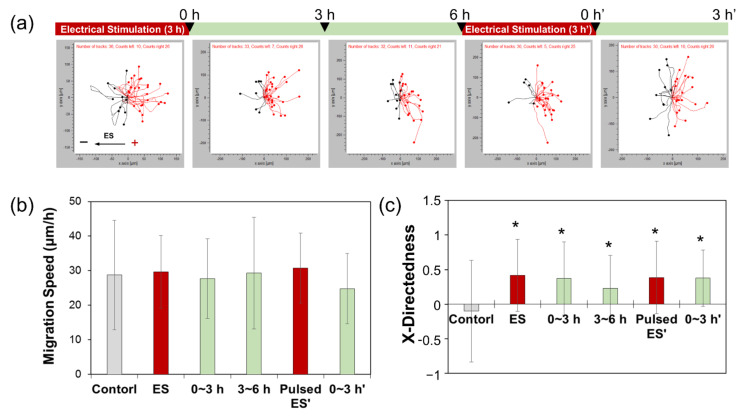
Migration assay of ADSCs subjected to 1200 μA double-round pulsed electrical stimulation for 3 h, at a 6 h interval. (**a**) Cell tracking data, (**b**) migration speed, and (**c**) X-directedness were determined for ADSCs subjected to 1200 μA electrical stimulation for 3 h, followed by 6 h rest and re-stimulation for 3 h. * represents *p* < 0.05 compared to controls not exposed to electric current.

To determine the appropriate level of ES for maintaining directional cell movement, we exposed cells to the same level of ES as used previously, but in an intermittent cycle by which 3 h exposure to electric current was followed by a 6 h rest period, after which the ES was repeated for 3 h. These intermittent repetitions of ES caused ADSCs to migrate directly toward the anode, as indicated by the migration trajectories (Figure 4a). Migration speed was not affected by the electric current or the number of exposure repetitions (Figure 4b). Directional migration toward the anode decreased once ES ceased; however, as soon as the repetitions of ES resumed, directional movement toward the anode increased again and continued even after ES was stopped (Figure 4c). In other words, although cell groups were exposed to ES for an equivalent time period (continuous ES for 6 h as opposed to two repetitions of 3 h ES each), the repetition of shorter bursts of ES was more effective in maintaining the cell migration direction without causing cell damage.

We had previously shown that human tonsil-, adipose tissue-, and bone marrow-derived mesenchymal stem cells (MSC) migrate directly toward the anode when exposed to an electric current [24,25]. Other published studies have also indicated that either direct or alternating current stimulate the directional migration of cells toward a specific cathodal or anodal orientation [23,36,37,38,39,40]. Adult human bone marrow-derived MSCs and induced pluripotent stem cells are stimulated toward strong anodal electrotaxis [33,41], whereas other cells, such as murine adipose stromal cells, and neural stem cells derived from human embryonic stem cells, responded toward the cathode when exposed to electric fields [42,43]. Therefore, it has been shown that different cell types, organisms and species—or the same test subjects exposed to different experimental conditions—show specific preference toward either cathodal or anodal electrotaxis during ES [36,44,45]. Although the effect exerted by electric fields of physiological strength on cell migration has been known for more than a decade, we now know that it is not simply a matter of all cells migrating by the same orientation. Research supports that electrotaxis can regulate cell positioning more precisely in multiple physiological situations. The migratory mechanism of mechanical stress has been widely studied but remains poorly understood. Various studies have reported that directional switching in electrotaxis could be determined by multiple signaling pathways such as GCase, PI3K, Ras, TORC2, PLCγ, and PLA2, which guide cell movement toward either the cathode or anode [46].

The application of a 1200 μA electric current for 3 h significantly increased the X-directedness of the cells in our study, whereby cells moved more directly toward the anode, indicating that these experimental conditions were sufficient to trigger electrotaxis. Moreover, directional cell migration was maintained for 6 h without any further exposure, following ES for 3 h. Based on these data, the ideal conditions for maintaining cell migration by pulsed ES were determined as 3 h stimulation followed by 6 h pause time.

**Figure 5:**
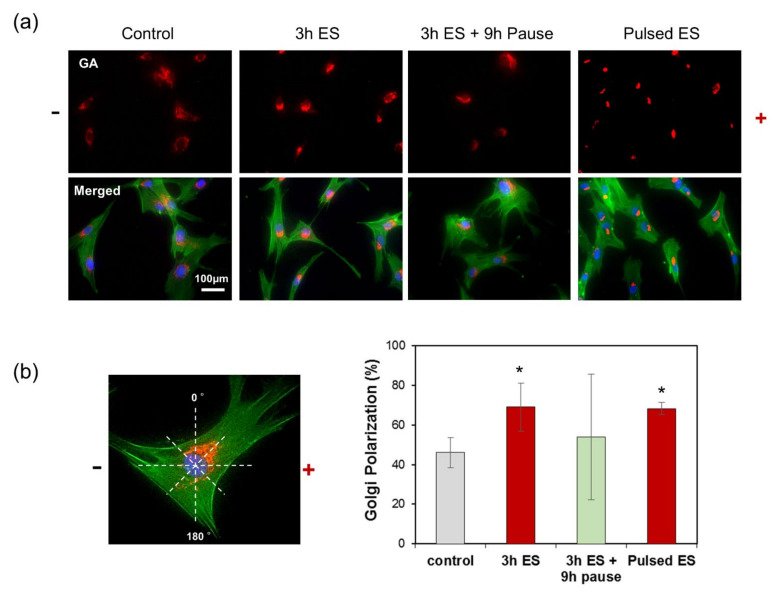
Golgi polarization of ADSCs under electrical stimulation. (**a**) Immunostaining of the actin cytoskeleton, nucleus, and Golgi apparatus of cells exposed and not exposed to electric signaling. Cells were fixed and triple-labelled with GM130 antibody (Golgi marker, red), Alexa Fluor 488-conjugated phalloidin (F-actin, green), and Hoechst #33258 (nucleus, blue). (**b**) The percentage of cells presenting with Golgi polarization towards the anode, counted in the 0–180° range. * represents *p* < 0.05 compared to controls not exposed to electric current.

Furthermore, we observed immunofluorescence images of the GA and actin cytoskeleton, as shown in Figure 5a, to determine whether pulsed ES affected Golgi polarization. In the absence of an electric current, the GA revealed its usual shape surrounding the nucleus and no polarized GA were present. In contrast, when cells were exposed to an electric current of 1200 μA for 3 h, the Golgi centroid was positioned in front of the nuclear centroid, along the direction of cell migration. In other words, the repositioning of the GA during polarization corresponded with the direction of cell migration—toward the anode—in response to ES. Polarized Golgi were observed, positioned in front of the nucleus parallel to the direction of cell migration, 9 h after the electric current was terminated. However, Golgi depolarization was also observed with spherical cytoskeleton deformation. Re-stimulation of cells for 3 h re-induced Golgi polarization toward the anode, in the direction of cell migration. To quantify the polarization of the GA, we counted the polarized Golgi in the range 0–180° when the anode was to the right (Figure 5b). The percentage of Golgi polarization in response to 1200 μA ES for 3 h was significantly increased, but slightly decreased in pulsed ES due to the dispersal of the GA in the absence of an electric signal. However, pulsed ES caused Golgi repolarization and reorientation toward the direction of cell migration.

Previous studies have shown that GA polarization plays an important role in directional cell migration during electrotaxis [27,28]. However, the molecules associated with the migration direction in electrotaxis have not been confirmed, and the underlying molecular mechanisms of these processes remain unclear [36,47]. Migrating cells become polarized toward the direction of movement, which occurs via reorientation of the cellular microtubule-organizing center, including the centrosome and GA. Among these, Golgi is involved in directional migration and cell polarity [48,49], and Golgi positioning has been related to the establishment and maintenance of cell polarity [50]. Our results confirmed that repeated ES created a stable condition for inducing directional migration toward the anode, with Golgi polarization.

## 4. Conclusions

Herein, we proposed electrotaxis induced by a pulsed current to reduce the risks to cells associated with ES and enhance the directionality of cell migration. In pulsed ES, directional migration toward the anode was maintained, regardless of the presence or absence of a direct current. Moreover, we confirmed that Golgi polarization is required to maintain directional migration in electrotaxis. Our study clearly demonstrated that the intermittent application of direct current can be an important key in controlling directional cell migration without inducing current-related cell death. Also, we suggest that exogenous ES may be an important tool to regulate homogenously cell infiltration into 3D tissue engineering construct for regenerative technology.

## Data Availability

The data presented in this study are available on request from the corresponding author.
